# Simulation of the flow field and the chemical reaction coupling of selective catalytic reduction (SCR) system using an orthogonal experiment

**DOI:** 10.1371/journal.pone.0216138

**Published:** 2019-07-12

**Authors:** Qihua Ma, Dongjian Zhang, Xuehui Gan

**Affiliations:** 1 State Key Laboratory for Modification of Chemical Fibers and Polymer Materials, Donghua University, Shanghai, China; 2 School of Mechanical and Automotive Engineering, Shanghai University of Engineering Science, Shanghai,China; 3 Key Laboratory of High Performance Fibers & Products, Ministry of Education, Donghua University, Shanghai,China; 4 Key Laboratory of Textile Science & Technology (Donghua University), Ministry of Education, Shanghai, China; Cranfield University, UNITED KINGDOM

## Abstract

It is difficult to simulate both the flow field and the chemical reaction using, respectively, the flow state and kinetics calculations and actually reflect the influence of the gas flow state on the chemical change in a selective catalytic reduction (SCR) system. In this study, the flow field and the chemical reaction were therefore coupled to simulate a full Cu-Zeolite SCR system and the boundary conditions of the simulation were set by a relevant diesel engine bench test which included the exhaust temperature, the mass flow, and the exhaust pressure. Then, the influence of the gas flow state on the NO_x_ conversion efficiency was investigated. Specifically, an orthogonal experimental design was used to study the influence of the injection parameters (position, angle, and speed) on the NH_3_ distribution by establishing the NH_3_ uniformity coefficient γ at the SCR catalyst capture surface in the flow field simulation. Then, the velocity capture surface of the SCR catalyst front section was sliced into coupled data transfer interfaces to study the effects of exhaust temperature, ammonia to NO_x_ ratio (ANR), and the NO_2_/NO_x_ on the NO_x_ conversion efficiency. This was used as guidelines to optimize the SCR system control strategy. The results showed that a 1150 mm injection position, a 45°injection angle, and a 23 m/s injection velocity provided the most uniform NH_3_ distribution on the SCR catalyst capture surface. For constant injection parameters, the NO_x_ conversion efficiency was the highest when the exhaust temperature was 200°C—400°C, the ANR was 1.1, and NO_2_/NO_x_ was 0.5.

## Introduction

Growing concerns about the environment have caused regulations concerning nitric oxides (NO_x_) to become increasingly stringent [1]. For instance, the NO_x_ emission limits of the European heavy-duty emission regulations have been reduced from 8.0 to 0.46 g/KWh [2]. Therefore, selective catalytic reduction (SCR) has become an essential after-treatment technique to ensure compliance with current stringent emission standards [3]. In this process, a urea-water solution (UWS) is injected into an exhaust pipe and reacts with NO_x_ to form nitrogen (N_2_) and water vapor (H_2_O). The use of a catalyst allows this reaction to proceed at a relatively low temperature. Under normal conditions, the UWS contains 32.5% urea and is injected into the exhaust pipe upstream of the SCR catalyst so that it interacts with hot exhaust gas to form gaseous urea. Then, the gaseous urea decomposes into ammonia (NH_3_) via thermolysis and hydrolysis, and the produced NH_3_ subsequently adsorbs to the SCR catalyst surface, and NO and NO_2_ directly react with the adsorbed NH_3_, via the Eley–Rideal mechanism [4].

Currently, the development of a diesel engine SCR system device is mainly based on simulations which are divided into two main categories: flow field simulations based on the flow state and chemical reaction simulations based on kinetics calculations. The flow field approach simulates the flow state of the exhaust gas in the exhaust pipe and helps not only to optimize a large number of design parameters of the SCR system, but also considerably shortens the turnaround time necessary to develop new products [5,6]. Considerable efforts have been made to model the SCR system using flow field simulations [7]. Wurzenberger et al. [8] performed a full flow field simulation of an HSO (Hydrolysis-SCR-Oxidizer Catalyst) system that consisted of urea injection, a homogeneous gas phase, and catalytic reactions. Benjamin et al. [9] performed a flow field study using a droplet model for urea injection and SCR reactions in a simple diffuser geometry. The flow field model was used to measure the impact of the injection velocity, spray angle, and droplet size on the overall performance of the SCR system in a study performed by Capetillo et al [10]. However, flow field simulations cannot accurately predict the chemical changes that occur in internal SCR catalysts because of the complicated gas flow state in the exhaust pipe. Therefore, chemical reaction simulations that use kinetics calculations are used to study the NO_x_ conversion efficiency of many individual chemical reactions [11,12].

Jinke et al. [13] created a chemical kinetics calculation model of an SCR catalyst to further study the effect of the reaction temperature, the space velocity, the ammonia-to-NO_x_ ratio (ANR), and the oxygen content on the NO_x_ conversion efficiency. Metkar et al. [14–17] used a more detailed chemical SCR system reaction model to determine the reaction and diffusion in the radial direction across a wash coat to obtain the Fe and Cu catalyst kinetic parameters. Using the chemical reaction simulation approach has many advantages, including a high accuracy and low time-consumption [18]. However, the boundary conditions consider only a uniform distribution, which does not accurately reflect the influence of the gas flow state on chemical changes for a complete simulation of an SCR system. Therefore, a combination of the two approaches would not only consider the flow state of the exhaust gas but would also be able to accurately calculate the chemical reaction of the SCR catalyst.

The study presented here proposes a method that couples the flow field and the chemical reaction methods to simulate a complete Cu-Zeolite SCR system to determine the influence of the gas flow state on the NO_x_ conversion efficiency. A bench test on a relevant diesel engine that included the exhaust temperature, the mass flow, the exhaust pressure, and the NO_x_ concentration was conducted to determine the boundary conditions for the different simulations. The flow field simulation was extended to study the quality of the injection parameters by establishing the NH_3_ uniformity coefficient γ on the capture surface of the SCR catalyst. The velocity of the SCR catalyst capture surface front section was sliced at data transfer interfaces of the flow field, and the chemical reaction was coupled to study the effects of the exhaust temperature, ANR, and NO_2_/NO_x_ on the NO_x_ conversion efficiency which provides guidelines to optimize the SCR system control strategy.

## Model and method

To use the coupling of the flow field and the chemical reaction to simulate an SCR system, each sub-process must be represented by a mathematical model. The accuracy of the simulation results depends on the accuracy of the mathematical model and the boundary conditions. The boundary conditions are measured via a bench test and the mathematical models adopted in this study for the sub-processes also have undergone maturation[19]. This section contains a detailed description of the mathematical models.

### 2.1 Flow field model

A Re-normalization Group Theory (RNG) *k*−*ε* model [20] is used to simulate the complicated turbulent process in the exhaust gas. It provides an analytical equation for the viscous flow, including the turbulent kinetic energy (*k*) equation and the dissipation of the turbulent kinetic energy(*ε*) equation. The transport equation for turbulent kinetic energy is given by:

$$\frac{\partial \left(\rho k\right)}{\partial t}+\frac{\partial \left(\rho u_{i}k\right)}{\partial x_{i}}=\sigma_{ij}\frac{\partial u_{i}}{\partial x_{j}}+\frac{\partial }{\partial x_{j}}\left(\frac{\mu }{{\mathrm{Pr}}_{k}}\frac{\partial k}{\partial x_{j}}\right)-\rho \varepsilon +S$$ (2.1)

Where *S* is the source term and the stress tensor *σ*_*ij*_ is given by:

$$\sigma_{ij}=2\mu_{t}S_{i}{}_{j}-\frac{2}{3}\delta_{ij}\left(\rho k+\mu_{t}\frac{\partial u_{i}}{\partial x_{i}}\right)$$ (2.2)

The turbulent viscosity *μ*_*t*_ is given by:

$$\mu_{t}=\rho C_{\mu }k^{2}/\varepsilon$$ (2.3)

The transport equation for turbulent dissipation is given by:

$$\frac{\partial (\rho \varepsilon )}{\partial t}+\frac{\partial \left(\rho u_{i}\varepsilon \right)}{\partial x_{i}}=\frac{\partial }{\partial x_{j}}\left(\frac{\mu }{Pr_{\varepsilon }}\frac{\partial \varepsilon }{\partial x_{j}}\right)+c_{\varepsilon 3}\rho \varepsilon \frac{\partial u_{i}}{\partial x_{i}}+\left(c_{\varepsilon 1}\frac{\partial u_{i}}{\partial x_{j}}\sigma_{ij}-c_{\varepsilon 2}\rho \varepsilon +c_{s}S\right)\frac{\varepsilon }{k}-\rho R$$ (2.4)

Where *ρ* is density, *u*_*i*_ is the velocity, Pr_*k*_, Pr_*ε*_ are the turbulent Prandtl number (0.7), *δ*_*ij*_ is the system number of Kronecher, *δ*_*ij*_ = 1(*i* = *j*), *δ*_*ij*_ = 0(*i*≠*j*), *S*_*ij*_ is the mean strain rate tensor, *C*_*μ*_, *c*_*ε*1_, *c*_*ε*2_ *c*_*ε*3_, and *c*_*S*_ are model constants equal to 0.0845, 1.42, 1.68, -1, and 0 respectively. *R* depends on the turbulence model and is defined by:

$$R=\frac{C_{\mu }\eta^{3}\left(1-\eta /\eta_{0}\right)}{1+\beta \eta^{3}}\frac{\varepsilon^{3}}{k}$$ (2.5)

For the RNG *k*−*ε* model, we set *η*_0_ to 4.38 in the initial conditions and *η* is given by:

$$\eta =\frac{k}{\varepsilon }\left|S_{ij}\right|$$ (2.6)

The discrete droplet model (DDM) [21] is used to simulate the UWS sprays and ignores the primary atomization process of the liquid phase. UWS is dosed in standard amounts (α), described as the feed ratio. Typically, a stoichiometric amount (α = 1) is used [22]. It considers that the UWS becomes a discrete droplet after leaving the nozzle with a trajectory solved by the Lagrange and the Euler methods. The Huh-Gosman breakup model [23] is used in the second breakup process of the droplet. The nozzle sprays the UWS into the exhaust pipe in the form of droplets and the atomization and diffusion of the droplets are caused by the higher exhaust temperature. Therefore, ammonia is generated from the evaporation of UWS through thermolysis and hydrolysis. The gaseous urea decomposes into ammonia (NH_3_) and isocyanic acid (HNCO):

$${\left(NH_{2}\right)}_{2}CO(g)\to NH_{3}(g)+HCNO(g)$$ (2.7)

HNCO will further react with H_2_O to form NH_3_ and carbon dioxide (CO_2_) through the hydrolysis process:

$$HNCO(g)+H_{2}O(g)\to NH_{3}(g)+CO_{2}(g)$$ (2.8)

For the homogeneous reactions in Eqs (2.7) and (2.8), the reaction rate *r* can be expressed by the Arrhenius Eq (2.9):

$$r=A\times e^{-E/RT_{g}}$$ (2.9)

Where *A* is pre-exponential factor, *T*_*g*_ is the exhaust temperature, *R* is the gas constant, and *E* is the activation energy. The kinetic parameters of the thermolysis and the hydrolysis reactions are listed in Table 1.

**Table 1:** Kinetic parameters of the thermolysis and hydrolysis reactions.

Reaction	Pre-exponential Factor(s^-1^)	Activation Energy(J/mol·k)
Thermolysis	1.27×10^4^	15540
Hydrolysis	1.13×10^10^	20980

## 2.2 Chemical reaction model

NH3 is mixed with the exhaust gas and enters the SCR catalytic reactor to undergo a catalytic reduction reaction. Selective catalytic reduction (SCR) of NO_x_ with NH_3_ mainly undergoes four chemical reactions:

$$4NH_{3}(g)+4NO(g)+O_{2}(g)\to 4N_{2}(g)+6H_{2}O(g)$$ (2.10)

$$2NH_{3}(g)+NO(g)+NO_{2}(g)\to 2N_{2}(g)+3H_{2}O(g)$$ (2.11)

$$8NH_{3}(g)+6NO_{2}(g)\to 7N_{2}(g)+12H_{2}O(g)$$ (2.12)

$$2NO_{2}\left(g\right)+2NH_{3}\left(g\right)\to N_{2}O\left(g\right)+N_{2}\left(g\right)+3H_{2}O\left(g\right)$$ (2.13)

Chemical reactions (2.10), (2.11), (2.12), (2.13) are the standard SCR, fast SCR, slow SCR, and the side reaction, respectively. The reaction rate *r* of SCR is also given by the Arrhenius equation. The pre-exponential factor *A* and activation energy *E* have been previously published by Atul Pant et al. [24] and are listed in Table 2.

**Table 2:** Kinetic parameters of the SCR chemical reaction.

Reaction	Pre-exponential Factor(s^-1^)	Activation Energy(J/mol·k)
NH3 adsorption	4.50	0
NH3 desorption	2.49×10^5^	9.75×10^4^
NH3 oxidation	1.39×10^6^	6.38×10^4^
Standard SCR	3.18×10^8^	8.80×10^4^
Fast SCR	2.33×10^7^	3.21×10^4^
Slow SCR	4.24×10^5^	5.83×10^4^
Side reaction	3.07×10^4^	4.82×10^4^

### 2.3 Coupling method

**Fig 1:**
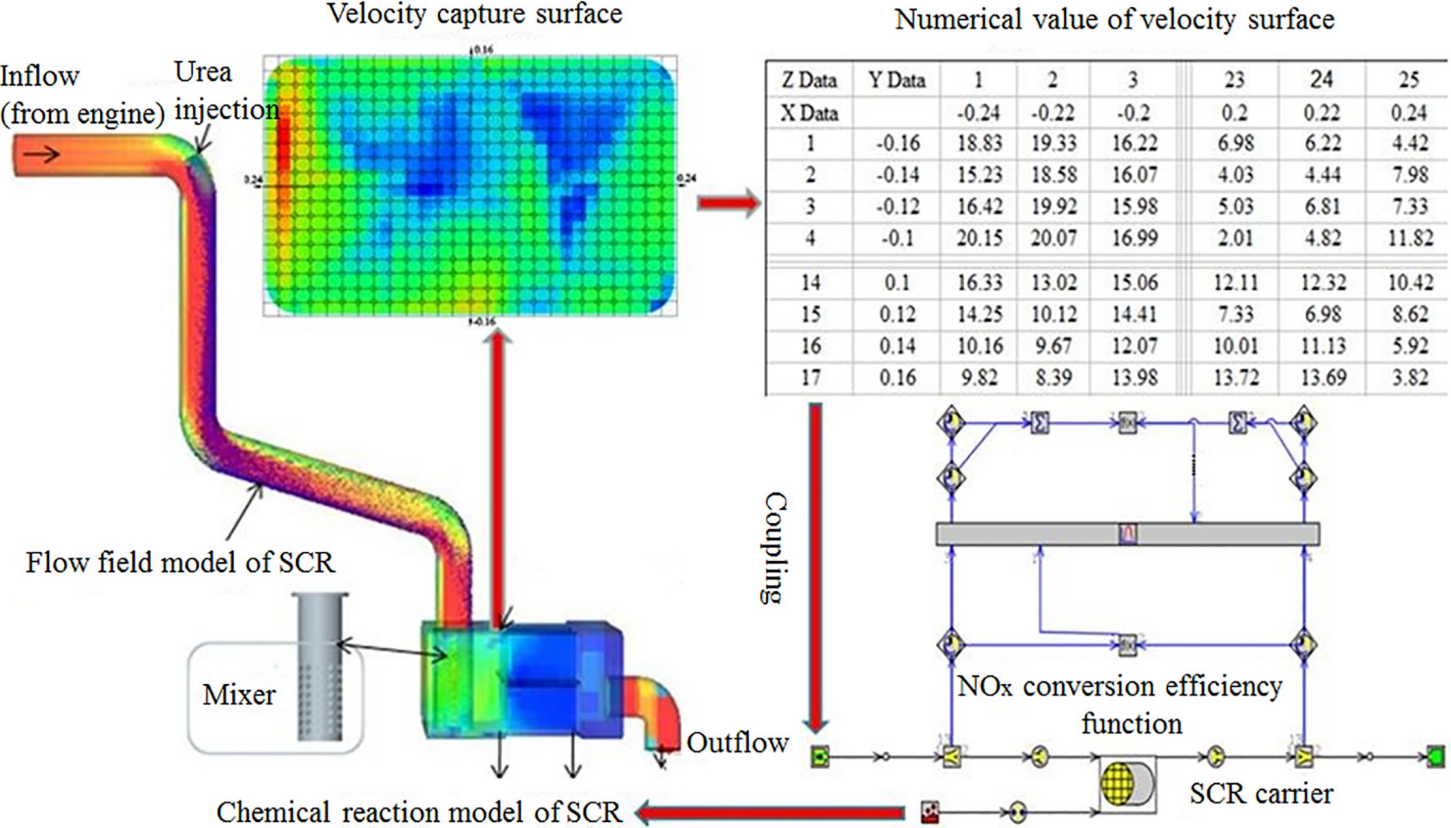
Flow field–chemical reaction coupling for the SCR system.

Hereby, we propose to couple the flow field model with the chemical reaction. The gas flow state of the SCR system is then calculated by the flow field model and the NO_x_ conversion efficiency of the SCR system is calculated by the chemical reaction model. Data transfer is achieved in the coupled calculation of the flow field and the chemical reaction. The specific coupling process occurs in several steps. First, the whole SCR system is calculated by the flow field simulation. After the calculation, software is used to process the simulation data and the velocity capture surface of the SCR catalyst front section is sliced. In this study the X-Y coordinate system is set up at the center of the velocity capture surface due to the large difference in velocity in the SCR catalyst front section and the velocity values of the 425 positions of the velocity capture surface are taken out and output into an Excel sheet. Then, the flow field model is coupled with the chemical reaction model of the SCR system through a user-defined function (UDF). There, the Excel sheets are read into the chemical reaction model of the SCR system. A schematic of the entire process is shown in Fig 1.

## Results and discussion

### 3.1 Bench test

To determine the emission parameters of a diesel engine as under different operating conditions, relevant engine bench tests were performed, and the basic parameters of the diesel engine are listed in Table 3. The power, torque, and speed of the diesel engine were measured using an AVL-ATA404 electric dynamometer, while NO_x_, CO, THC, and O_2_ gas concentrations were measured using an AVL-i60 and an AVL-PEU. Various sensors were used to measure the temperature, pressure, mass flow, and other physical parameters for each of the working conditions. A bench test was performed according to the requirements of the GB-17691-2005 emission limits and the measurement methods for automotive compression ignition, gas fuel ignition engines, and vehicle exhaust pollutants (China III, IV, V).

**Table 3:** Main technical parameters of the diesel engine.

Characteristic	Value
Air Intake method	Turbocharged intercooler
Cylinder number	6
Compression ratio	17:1
ignition order	1-5-3-6-2-4
Bore x stroke (mm)	126 x 155
Displacement (L)	11.596
Rated power (kW)	353

Previous research [25] has shown that the exhaust temperature is the main factor that affects the NO_x_ conversion efficiency in an SCR system. Therefore, a typical working condition of the diesel engine was selected where the working conditions were measured at 1000 rpm by adjusting the engine load and taking a 50 ^o^C exhaust temperature as the step. The exhaust parameters were used for the computational fluid dynamics (CFD) simulation boundary conditions of the SCR system, as reported in Table 4.

**Table 4:** The exhaust parameters of each working condition.

Working condition	Exhaust temperature (^o^C)	Mass flow (g/s)	Exhaust pressure (Pa)
1	100	62	100102
2	150	78	100185
3	200	90	100242
4	250	97	100335
5	300	105	100449
6	350	120	100660
7	400	147	101053
8	450	193	101807
9	500	220	102387
10	550	245	103124

The initial condition for each gas component proportion in the flow field and chemical reaction coupling calculation is shown in Table 5.

**Table 5:** Initial condition for each gas component.

Gas component	Proportion
CO_2_	17.13939%
N_2_	69.34727%
O_2_	5.63727%
H_2_O	7.82864%
NO	0.02976%
NO_2_	0.00766%

### 3.2 Establishment of the NH_3_ uniformity coefficient

**Fig 2:**
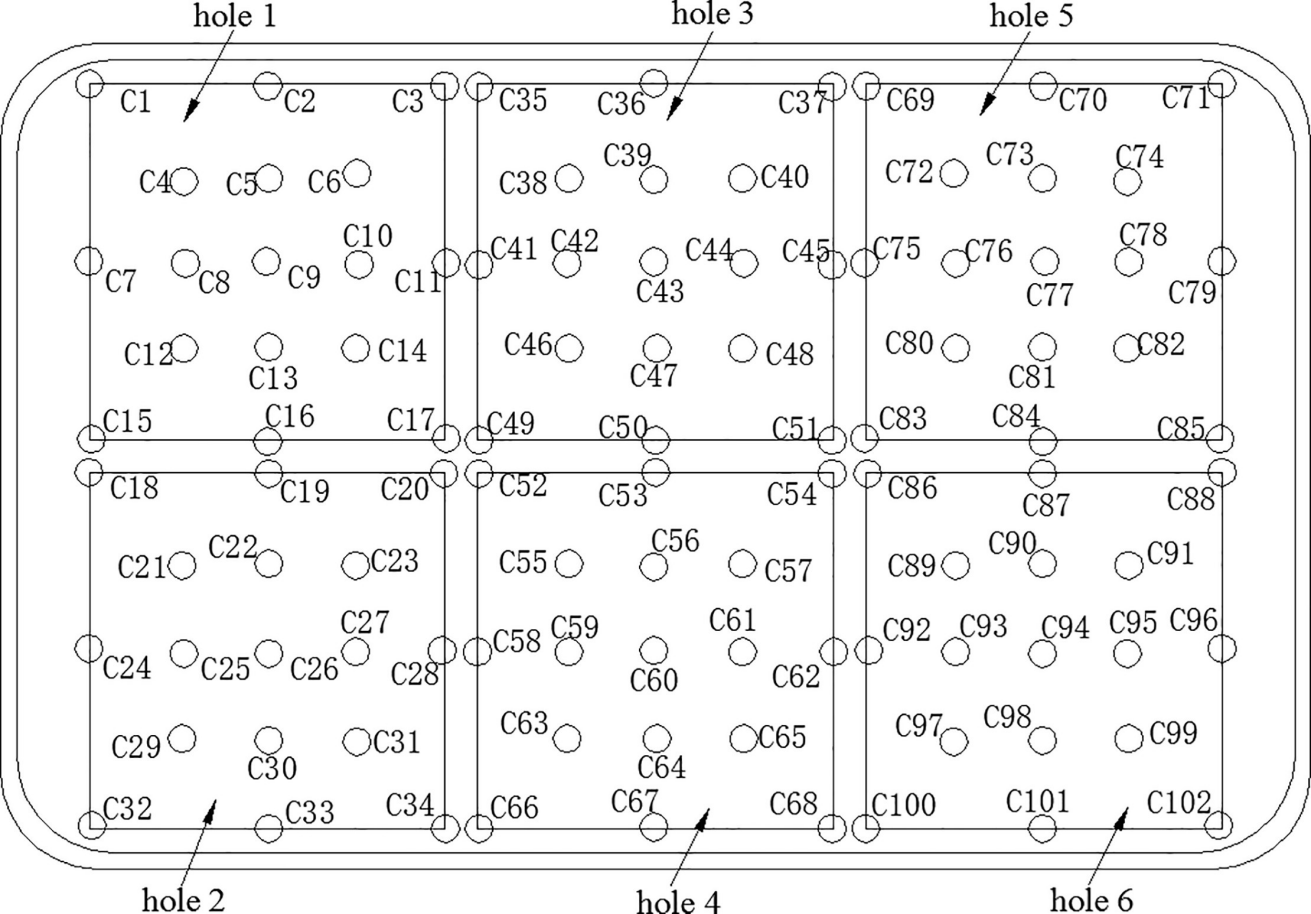
Selection point for NH_3_ concentration and velocity distribution on the SCR catalyst capture surface.

The uniformity coefficient represents the distribution of NH_3_ on the capture surface of the SCR catalyst and is well-correlated to the performance of SCR systems [22]. A high uniformity coefficient indicates that the NO_x_ conversion efficiency has been maximized and that the NH_3_ slip is minimized. The coefficient of variation (C.V) is introduced to measure the discrete degree of NH_3_ on the SCR catalyst capture surface and the NH_3_ uniformity coefficient γ is defined as:

$$\gamma =1-\frac{\sqrt{\frac{\sum_{i=1}^{n}{\left(c_{i}-\bar{c}\right)}^{2}}{n}}}{\bar{c}}$$ (3.1)

where *n* is the total number of points on the SCR catalyst capture surface, *c*_i_ represents the NH_3_ concentration, and $\bar{c}$ represents the average NH_3_ concentration at the capture surface. An NH_3_ uniformity coefficient γ near 1 indicates a uniform NH_3_ distribution on the capture surface. The uniformity of NH_3_ is calculated by considering 102 positions on the SCR catalyst capture surface, as shown in Fig 2.

### 3.3 Orthogonal design and analysis of the injection parameters

Orthogonal testing is a method used to make optimal selections based on mathematical statistics. In a multi-factor optimization test, mathematical statistics and the orthogonal principle are used to select representative and typical points from many test points. The experiment is arranged scientifically and reasonably using an "orthogonal table," and the optimal test results are obtained from a minimum number of test times. In this study, the orthogonal experimental design method was used to optimize the urea injection parameters in the SCR system. An analysis of the NH_3_ distribution uniformity at the SCR catalyst capture surface under different injection parameters, allowed the optimum combination of urea injection parameters to be determined.

Many physical and chemical processes occur between the urea injection and the SCR catalyst, including urea atomization, urea evaporation, thermolysis, and hydrolysis. The urea injection boundary conditions were the use of a single nozzle with a diameter of 0.78 mm. Simultaneously, to simulate an SCR system common working state, the exhaust parameters at 1000 rpm under a full load were selected as boundary conditions for the flow field simulation analysis.

#### 3.3.1 Orthogonal experimental design of injection parameters

**Fig 3:**
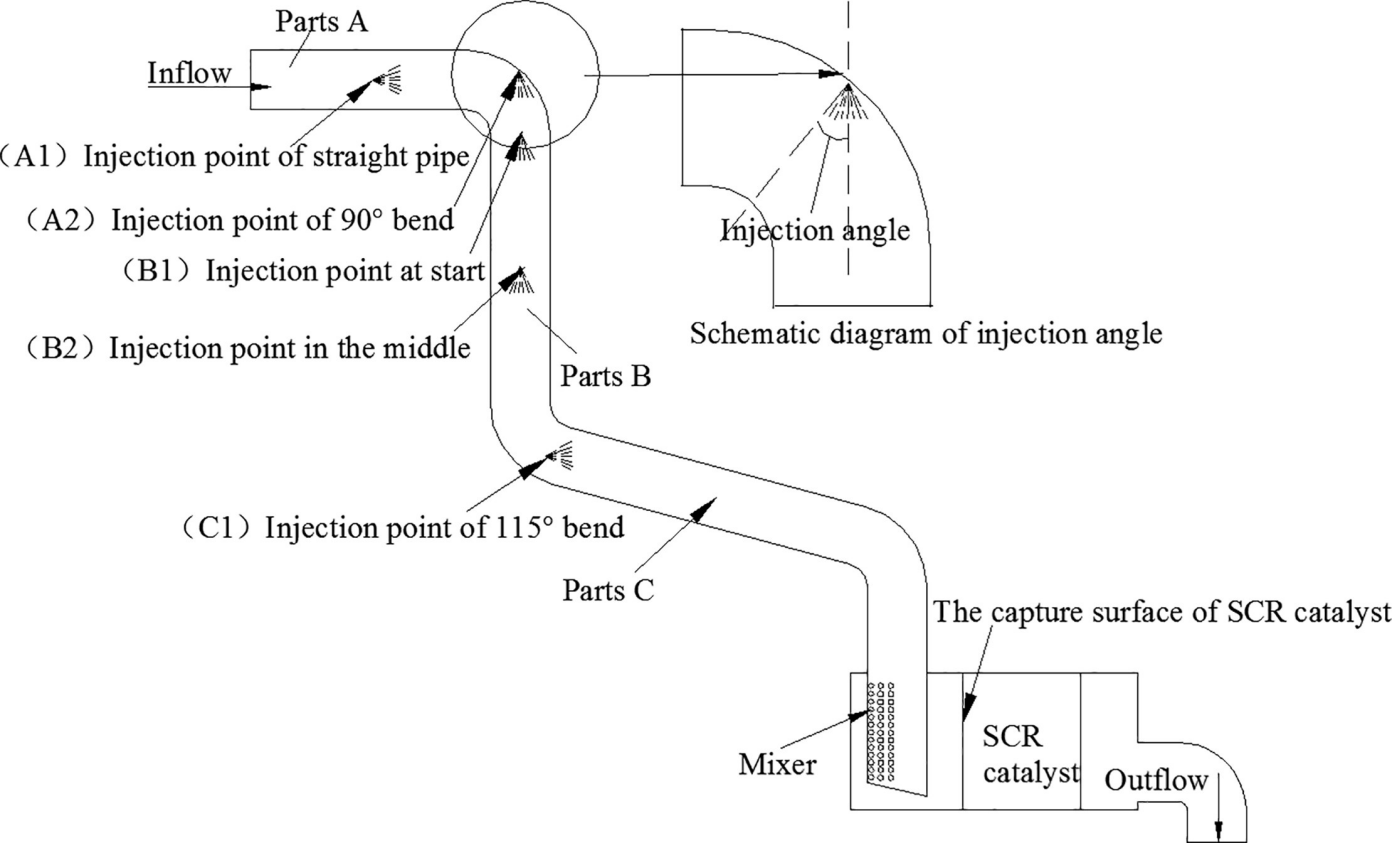
Urea injection scheme.

To obtain the best combination of the three factors affecting the NH_3_ uniformity on the SCR catalyst capture surface, 3 main factors were determined: A (the injection position), B (the injection angle), and C (the injection speed). The overall injection scheme is shown in Fig 3.

The index of the orthogonal experiment is the NH_3_ uniformity coefficient γ at the SCR catalyst capture surface. According to experiments, 3–5 levels are suitable to minimize the number of experiments, and therefore, 4 levels of experimental factors were chosen, as listed in Table 6.

**Table 6:** Factors and levels.

Level number	Factors		
	A Injection position (mm)	B Injection angle (deg)	C Injection velocity (m/s)
1	400	15	20
2	650	25	23
3	900	35	26
4	1150	45	29

#### 3.3.2 Orthogonal experiment analysis of the injection parameters

The orthogonal experiments were analyzed by L16 (43), and the effects of the different factors and levels on the NH_3_ uniformity coefficient γ at the SCR catalyst capture surface are listed in Table 7.

**Table 7:** Orthogonal test analysis.

Test number	Factors			Index
	A Injection position (mm)	B Injection angle (deg)	C Injection velocity (m/s)	NH_3_ uniformity coefficient γ
1	1	1	1	0.743
2	1	2	2	0.748
3	1	3	3	0.754
4	1	4	4	0.758
5	2	1	2	0.832
6	2	2	1	0.835
7	2	3	4	0.838
8	2	4	3	0.844
9	3	1	3	0.885
10	3	2	4	0.889
11	3	3	1	0.892
12	3	4	2	0.897
13	4	1	4	0.938
14	4	2	3	0.945
15	4	3	2	0.950
16	4	4	1	0.953
K_1_	3.003	3.398	3.423	-
K_2_	3.349	3.417	3.427	-
K_3_	3.563	3.425	3.428	-
K_4_	3.786	3.452	3.423	-
k_1_	0.751	0.850	0.856	-
k_2_	0.837	0.854	0.857	-
k_3_	0.891	0.856	0.857	-
k_4_	0.947	0.863	0.856	-
R	0.196	0.013	0.001	-

The advantage of using an orthogonal experiment is that complex multi-factor data processing problems are transformed into simple single-factor data processing problems. Therefore, each factor can be estimated to determine the best combination of urea ejection parameters in the SCR system just by calculating the orthogonal experimental data. *K*_1_, *k*_1_, *K*_2_, *k*_2_, *K*_3_, *k*_3_, *K*_4_, *k*_4_, and R in Table 2 are determined respectively as:

$$K_{1A}=0.743+0.748+0.754+0.758=3.003$$

$$K_{2A}=0.832+0.835+0.838+0.844=3.349$$

$$K_{3A}=0.885+0.889+0.892+0.897=3.563$$

$$K_{4A}=0.938+0.945+0.950+0.953=3.786$$

In the formula, *K*_1A_, *K*_2A_, *K*_3A_, and *K*_4A_, indicate the sum of the test results of factor A using 1, 2, 3, and 4 levels, respectively. To compare the injection quality of different A levels, especially in experiments with unequal factors, the *k* value is introduced:

k1A=K1A4=0.751;k2A=K2A4=0.837

k3A=K3A4=0.891;k4A=K4A4=0.947

**Fig 4:**
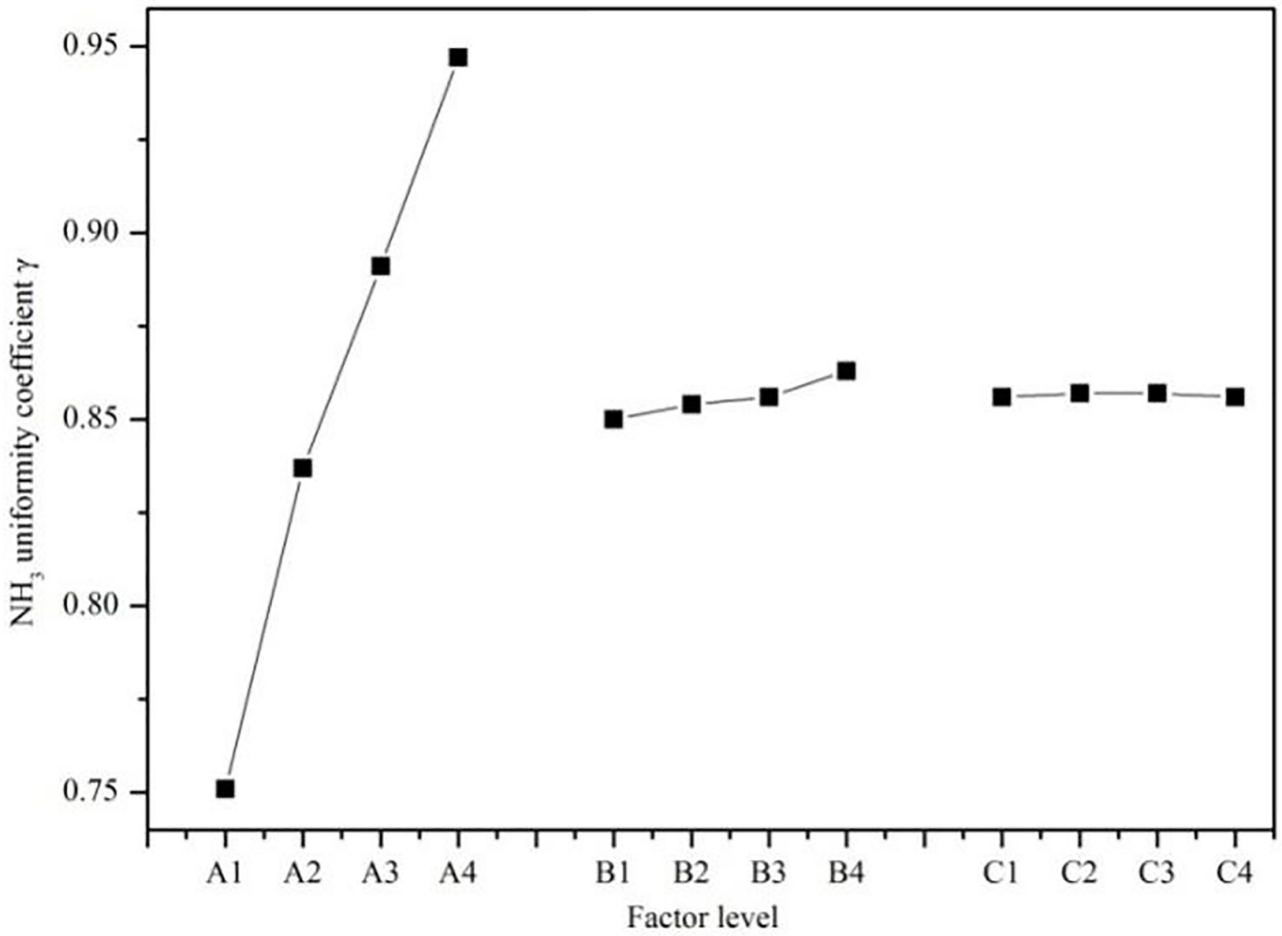
Relationship between factors and the index.

In the formula, *k*_1A_, *k*_2A_, *k*_3A_, and *k*_4A_ represent the average NH_3_ uniformity coefficient γ on the SCR catalyst capture surface of each level of factor A, respectively. Similarly, the three remaining columns of *K*_1_, *K*_2_, *K*_3_, and *K*_4_ (or the averages *k*_1_, *k*_2_, *k*_3_, and *k*_4_) can then be calculated. To determine how various factors directly influence the NH_3_ uniformity, the factor level is taken as the horizontal coordinate, while the average index value is taken as the ordinate. The relationship between the factor and the index is represented in Fig 4.

To illustrate the impact of different factor levels on NH_3_ uniformity, the horizontal coordinate was chosen to be the factor level and the ordinate was set as the NH_3_ uniformity coefficient γ. Fig 4 shows that changes in the A factor level lead to a large change relative to other factors in γ, so A is clearly the main factor that affects the NH_3_ uniformity on the SCR catalyst capture surface. To obtain a quantitative expression, the degree of dispersion is described using the extreme value, *R* which is obtained by subtracting the minimum value from the maximum value of the *k*_1_, *k*_2_, *k*_3_, and *k*_4_ values of each column. Therefore, *R* = *k*_max_−*k*_min_.

*R* reflects how strongly each factor influences the index in the orthogonal experiment, where a high *R* value indicates that a factor strongly influences the index. According to Table 6, the main and secondary factors are A-B-C. The greater the value of γ at the SCR catalyst capture surface, the better the injection quality, and consequently, the best combination is A_4_B_4_C_2_.

### 3.4 Changes in velocity distribution at different exhaust temperatures

The SCR mixer was designed based on the previously-reported results of Dehui et al. [26], and the opening angle of the porous tube was selected to be 360 degrees, 8 rows, and 8 columns. The holes correspond to 30 degrees per column, and there were 64 holes with a perforation rate of 1.44, as shown in Fig 1. A 1150-mm injection position, a 45^o^ injection angle, and a 23 m/s injection velocity was shown to produce the most uniform NH_3_ distribution at the SCR catalyst capture surface, according to the values listed in Table 6. These values provide a basis to perform the coupling study of the flow field and the chemical reaction in the SCR system.

**Fig 5:**
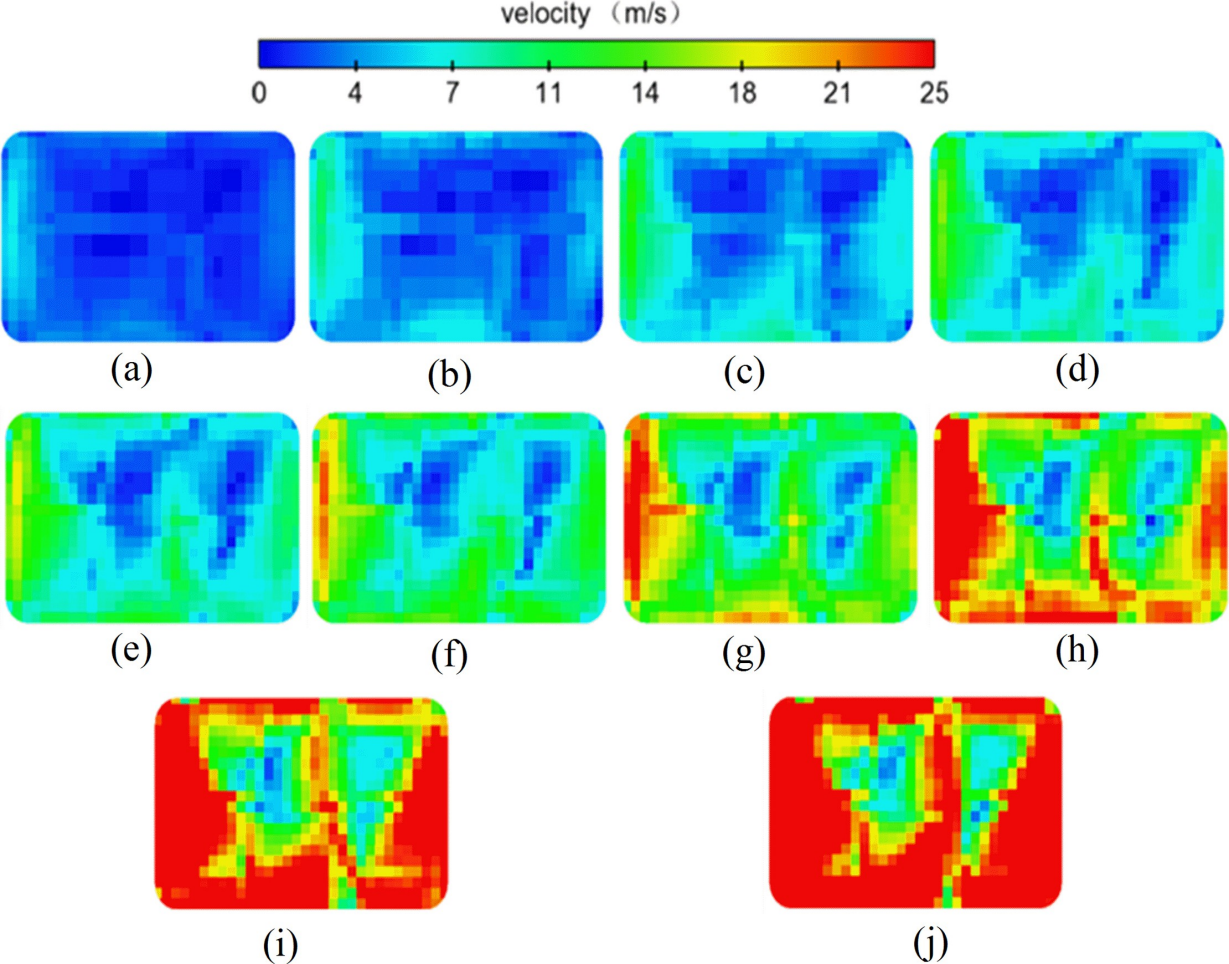
Flow velocity distribution at the front of the catalyst. (Charts a, b, c, d, e, f, g, h, i and j show that the exhaust temperature is 100°C, 150°C, 200°C, 250°C, 300°C, 350°C, 400°C, 450°C, 500°C and 550°C respectively.).

**Fig 6:**
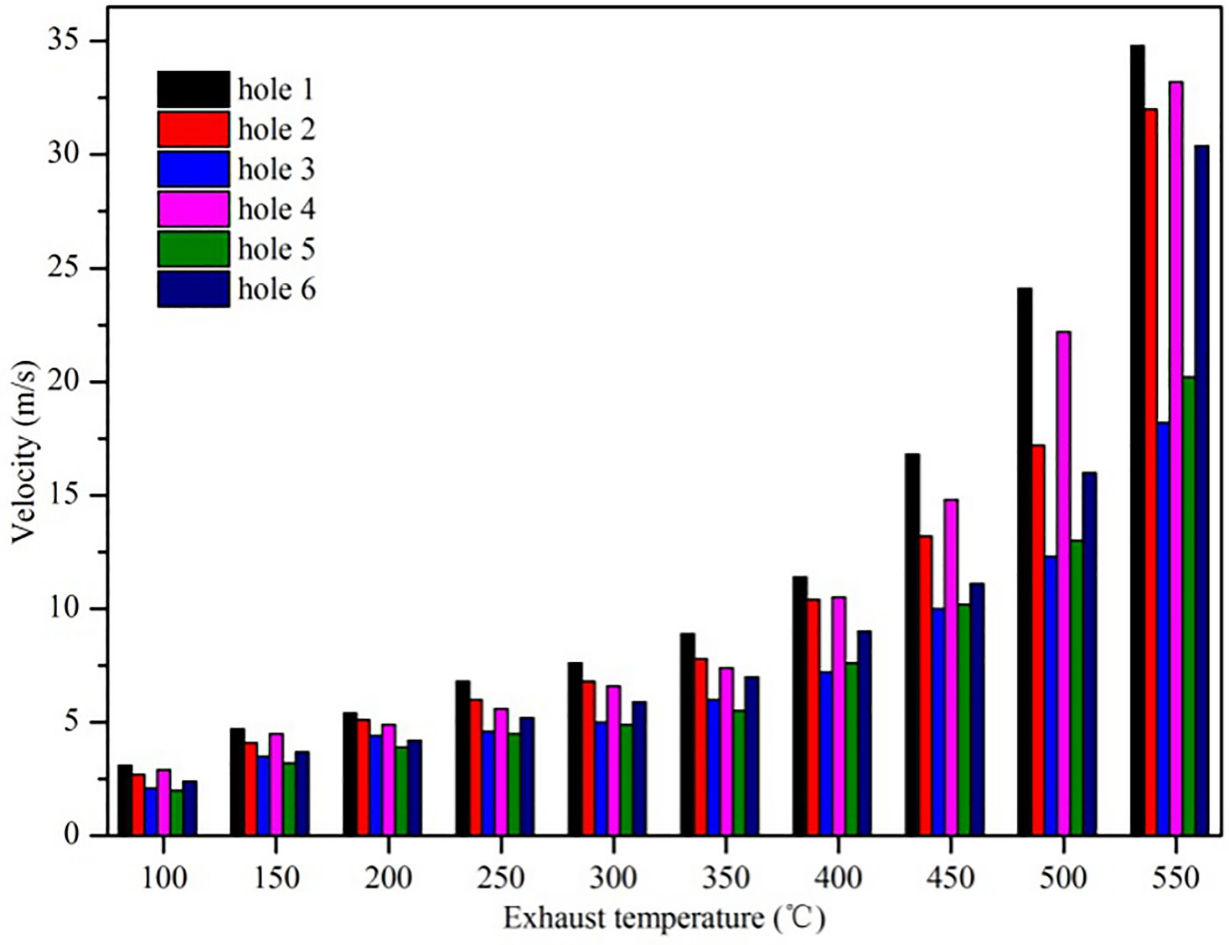
Velocity change.

When the exhaust temperature increases, the turbulent flow becomes more complex, and the velocity at the SCR catalyst capture surface shows an obvious difference, as shown in Fig 5. The velocity becomes less uniform at higher exhaust temperatures. Fig 6 shows a quantitative analysis of the average flow velocity in the six holes on the capture surface of the SCR catalyst. The hole velocity difference gradually increases as the exhaust temperature increases. The flow velocity of holes 1, 2, 4, and 6 show large increases because the air flow is dispersed and disturbed as the air flow passes through the SCR mixer and a certain aggregation occurs in holes 1, 2, 4, and 6. The flow velocity is too high in these holes and too low in others which causes an inconsistent chemical reaction time of the SCR system that affects the NO_x_ conversion efficiency within the various holes.

Therefore, the change in the diesel exhaust temperature directly affects the velocity uniformity at the SCR catalyst capture surface and further affects the NO_x_ conversion efficiency in the SCR system.

### 3.5 NO_x_ conversion efficiency study

The results above show obvious differences in the exhaust temperatures of the diesel engine results in differences in the flow velocity at the SCR catalyst capture surface, which leads to differences in the chemical reaction time that further affects the NO_x_ conversion efficiency. The traditional method where a mean one-dimensional chemical reaction is used cannot accurately reflect the influence of the flow velocity movement in the SCR system on the chemical reaction. Therefore, the flow field and chemical reaction coupling method was adopted to study of the effect of exhaust temperature, ammonia-to-NO_x_ ratio (ANR), and NO_2_/NO_x_ on the NO_x_ conversion efficiency.

#### 3.5.1 Exhaust temperature

**Fig 7:**
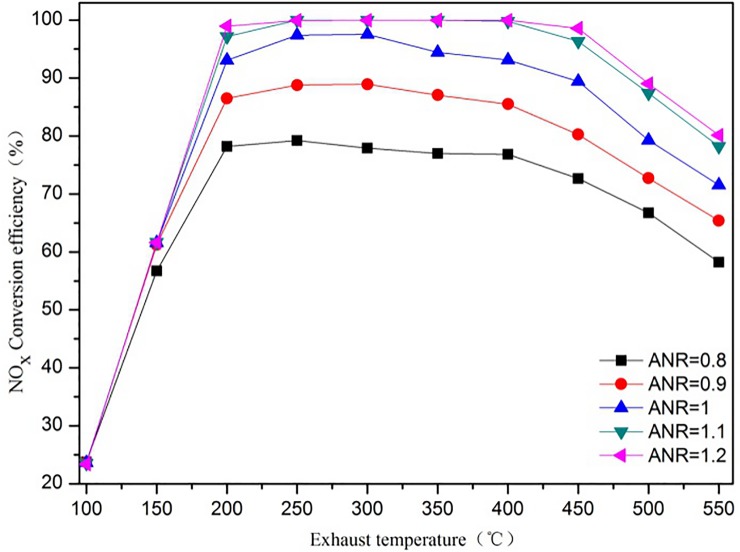
The effect of the exhaust temperature on NO_x_ conversion efficiency at NO_2_/NO_x_ = 0.1.

The exhaust temperature greatly influences the SCR catalyst activity, and higher temperatures, increase both the SCR catalyst activity and the NO_x_ conversion efficiency. Fig 7 clearly shows that the exhaust temperature affects the NO_x_ conversion efficiency, and that when the exhaust temperature increases, the NO_x_ conversion efficiency generally increases at different ANR. When ANR = 1.2 and the exhaust temperature is 450 ^o^C, the NO_x_ conversion efficiency reaches a maximum of 99%. When the exhaust temperature is 100 ^o^C, the NO_x_ conversion efficiency is only 23% because the SCR catalyst activity and the chemical reaction rate are both low. It is worth noting that the NO_x_ conversion efficiency increases with the temperature (100°C—200°C). When ANR = 1.2, the NO_x_ conversion efficiency at 200 ^o^C increases by nearly 426% compared to 100 ^o^C. The NO_x_ conversion efficiency tends to remain stable in the 200°C—400°C exhaust temperature range, but when the exhaust temperature is above 400°C, the NO_x_ conversion efficiency slightly decreases.

#### 3.5.2 Ammonia to NO_x_ ratio (ANR)

**Fig 8:**
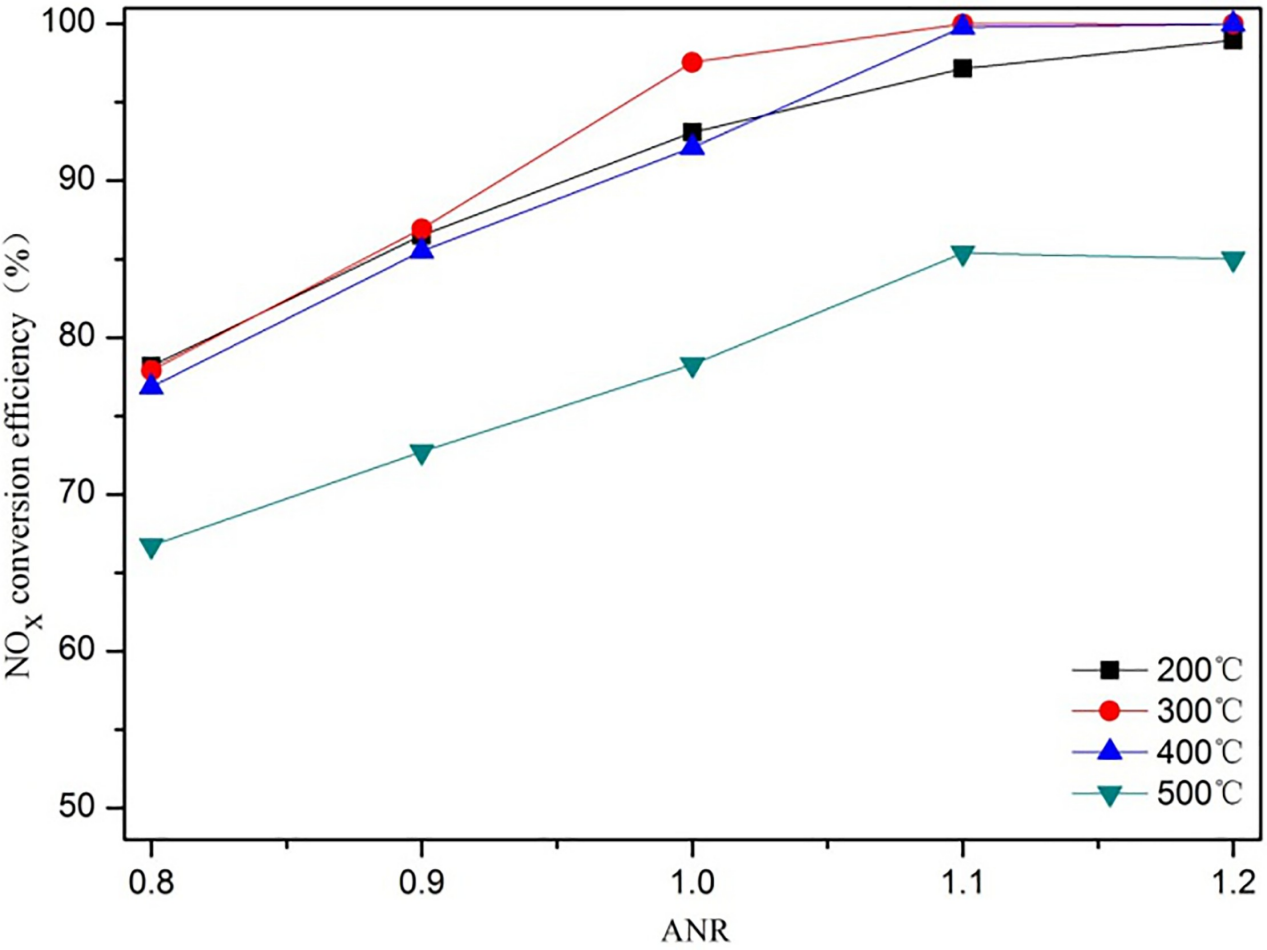
The effect of the ANR on the NO_x_ conversion efficiency at NO_2_/NO_x_ = 0.1.

The ammonia to NO_x_ ratio (ANR) is the molar ratio of NH_3_ to NO_x_ in the exhaust that assumes that 1 mol NO_x_ needs 1 mol NH_3_ in the SCR chemical reaction, and the influence of the ANR on the NO_x_ conversion efficiency is shown in Fig 8. As the ANR increases, the NO_x_ conversion efficiency at different exhaust temperatures improves, reaching a value of 99% at an exhaust temperature of 400 ^o^C. When ANR = 0.8, the NO_x_ conversion efficiency is generally low at different exhaust temperatures because the molar content of NH_3_ is too low, which means that the NO_x_ has not fully reacted, and a NO_x_ conversion efficiency of only 63% is obtained at 500 ^o^C. When the ANR is between 0.8 and 1.1, the NO_x_ conversion efficiency increases as the ANR increases, but since NH_3_ is partially adsorbed on the SCR catalyst wall, the NO_x_ conversion efficiency does not reach a maximum when the ANR = 1. Only when the ANR is between 1.1 and 1.2 does the NO_x_ conversion efficiency curve tend to become flat. In addition, no matter how high the ANR reaches, the NO_x_ conversion efficiency is generally low at an exhaust temperature of 500 ^o^C, which indicates that side reactions are prominent and secondary pollution is severe.

#### 3.5.3 NO_2_/NO_x_

**Fig 9:**
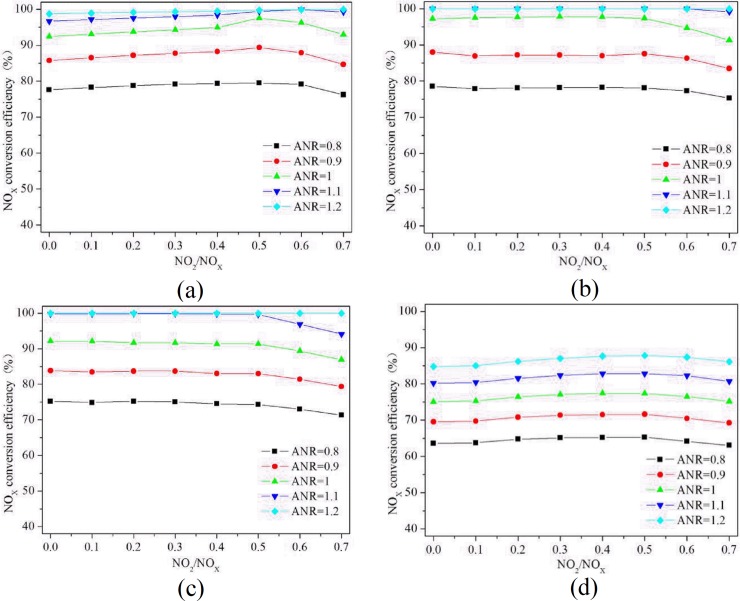
The effect of NO_2_/NO_x_ on the NO_x_ conversion efficiency. (Charts a, b, c and d show that the exhaust temperature is 200°C, 300°C, 400°C, 500°Crespectively.).

Equation (9) shows that the standard SCR chemical reaction (10) occurs almost 17 times more quickly than the slow SCR chemical reaction (12) at low temperatures [27–28]. In addition, if enough NH_3_ is present, it will react with NO and NO_2_ before undergoing the slow SCR chemical reaction (12). The influence of NO_2_/NO_x_ on the NO_x_ conversion efficiency is shown in Fig 9. When the exhaust temperature is between 200 ^o^C and 400 ^o^C and the ANR is between 1.1 and 1.2, the NO_x_ conversion efficiency remains steady and maximal as the NO_2_/NO_x_ increases. When the ANR is less than or equal to 1, the NO_x_ conversion efficiency decreases when the NO_2_/NO_x_ is greater than 0.5 because the excess NO_2_ will directly react with NH_3_ when NO_2_/NO_x_ exceeds 0.5 via the side reaction (13) with a small reaction rate, which decreases the NO_x_ conversion efficiency. When the exhaust temperature is 500 ^o^C, the NO_x_ conversion efficiency for different ANR values is lower than at other exhaust temperatures because N_2_O is produced as a by-product at exhaust temperatures above 500 ^o^C.

## Conclusions

A method that couples the flow field and the chemical reaction to simulate a full Cu-Zeolite SCR system is proposed in this report. In addition to studying the quality of the injection parameters by establishing the NH_3_ uniformity coefficient γ at the SCR catalyst capture surface in a flow field simulation using an orthogonal experimental design, the velocity capture surface at the SCR catalyst front section was sliced at the data transfer interfaces. Then, to provide guidelines for optimization of SCR system control strategy, the effects of the exhaust temperature, ANR, and NO_2_/NO_x_ ratio on the NO_x_ conversion efficiency were investigated, which allowed the following main conclusions to be drawn:

1. The different parameters were shown to have a great influence on the NH_3_ distribution uniformity on the capture surface of the SCR catalyst. When the injection position was placed closer to the SCR catalyst at a lower injection angle, the NH_3_ uniformity coefficient γ at the SCR catalyst capture surface decreased. The injection velocity was shown to have little effect on the NH_3_ distribution uniformity. Consequently, a 1150-mm injection position, a 45^o^ injection angle, and a 23 m/s injection velocity were shown to be the most suitable parameters for the SCR system.
2. At an identical speed, increasing the load, exhaust temperature, mass flow, and exhaust pressure caused a more complex turbulent flow. The flow velocity in the SCR catalyst capture surface was dispersed and disturbed because it was influenced by the mixer. The velocity distribution at the SCR catalyst capture surface is different, which leads to an inconsistent catalytic reduction reaction which further affects the NO_x_ conversion efficiency.
3. As the exhaust temperature increases, the NO_x_ conversion efficiency rapidly increases before becoming stable. When the exhaust temperature increases to 400 ^o^C, the NO_x_ conversion efficiency decreases slightly. Increasing the ANR and NO_2_/NO_x_ improves the NO_x_ conversion efficiency of the SCR system. The NO_x_ conversion efficiency is the highest at exhaust temperatures between 200 ^o^C and 400 ^o^C when the ANR is 1.1 and the NO_2_/NO_x_ ratio is 0.5.

## Supporting information

S1 Fig Analysis results of urea injection angle. (TIF)

S2 Fig Pressure drop diagram of SCR system. (TIF)

S1 File The effect of NO2NOx on the NOx conversion efficiency(minimal data set). (XLSX)

## Nomenclature

*σ_ij_*: stress tensor [Pa]
*μ_t_*: turbulent viscosity [Pa^.^s]
*u_i_*: velocity [m/s]
Pr_*k*_,Pr_*ε*_: turbulent Prandtl number (0.7)
*δ_ij_*: system number of Kronecker
*S_ij_*: mean strain rate tensor [Pa/s]
*c*_*μ*_,*c*_*ε*1_,*c*_*ε*2_: model constants equal to 0.0845, 1.42, and 1.68
*c*_*ε*1_,*c*_*S*_: model constants equal to -1 and 0
*T_g_*: exhaust temperature [K]
*E*: activation energy [J/mol^.^k]
*A*: pre-exponential factor [s^-1^]
*ρ*: density [kg^.^m^-3^]
*k*: turbulent kinetic energy [m^2.^s^−2^]
*ε*: turbulence dissipation rate [m^2.^s^−3^]
*γ*: NH_3_ uniformity coefficient
*c*: NH_3_ concentration

## References

[1] Aberg A, Widd A, Abildskov J, et al Parameter estimation and analysis of an automotive heavy-duty SCR catalyst model[J]. Chemical Engineering Science, 2017, 161:167–177.

[2] Yuan X, Liu H, Gao Y. Diesel Engine SCR Control: Current Development and Future Challenges[J]. Emission Control Science & Technology, 2015, 1(2):121–133.

[3] Johnson T V. Review of Diesel Emissions and Control[J]. International Journal of Engine Research, 2010, 10(5):275–285.

[4] Nova I, Lietti L, Tronconi E, et al Dynamics of SCR reaction over a TiO2-supported vanadia–tungsta commercial catalyst[J]. Catalysis Today, 2000, 60(1–2):73–82.

[5] Sturgess M P, Benjamin S F, Roberts C A. Spatial conversion profiles within a SCR in a test exhaust system with injection of ammonia gas modeled in CFD using the porous medium approach[C]//. Powertrains Fuels and Lubricants Meeting San Diego, Ca. SAE 2010.

[6] Abidin Z, Das K, Roberts C. 3D-Semi 1D Coupling for a Complete Simulation of an SCR System[J]. SAE, 2013.

[7] Paramadayalan T, Pant A. Selective catalytic reduction converter design: The effect of ammonia non-uniformity at inlet[J]. Korean Journal of Chemical Engineering, 2013, 30(12):2170–2177.

[8] Wurzenberger J. C. and Wanker R., Multi-scale SCR modeling, 1D kinetic analysis and 3D system simulation, SAE 2005-01-0948 (2005).

[9] Benjamin S F, Roberts C A. The porous medium approach applied to CFD modeling of SCR in an automotive exhaust with injection of urea droplets[C]// IMECHE Conference Internal Combustion Engines: Performance Fuel Economy and Emissions Conference. 2007.

[10] Capetillo A, Ibarra F. Multiphase injector modeling for automotive SCR systems: A full factorial design of experiment and optimization[J]. Computers & Mathematics with Applications, 2017, 74(1): 188–200.

[11] Konstandopoulos A G, Kostoglou M, Beatrice C, et al Impact of Combination of EGR, SCR, and DPF Technologies for the Low-Emission Rail Diesel Engines[J]. Emission Control Science & Technology, 2015, 1(3):213–225.

[12] Aberg A, Widd A, Abildskov J, et al Estimation of Kinetic Parameters in an Automotive SCR Catalyst Model[J]. Topics in Catalysis, 2016, 59(10–12):945–951.

[13] Jinke Gong, Jun Bao, Ligang Tan, et al Study on the characteristics of low temperature expression of urea SCR system [J]. Journal of Hunan University, 2013, 40 (1): 38–42.

[14] Metkar P S, Balakotaiah V, Harold M P. Experimental study of mass transfer limitations in Fe- and Cu-zeolite-based NH 3 -SCR monolithic catalysts[J]. Chemical Engineering Science, 2011, 66(21):5192–5203.

[15] Metkar P S, Balakotaiah V, Harold M P. Experimental and kinetic modeling study of NO oxidation: Comparison of Fe and Cu-zeolite catalysts[J]. Catalysis Today, 2012, 184(1):115–128.

[16] Metkar P S, Harold M P, Balakotaiah V. Selective catalytic reduction of NO x, on combined Fe- and Cu-zeolite monolithic catalysts: Sequential and dual layer configurations[J]. Applied Catalysis B Environmental, 2012, 111–112(1):67–80.

[17] Metkar P S, Harold M P, Balakotaiah V. Experimental and kinetic modeling study of NH 3 -SCR of NO x, on Fe-ZSM-5, Cu-chabazite and combined Fe- and Cu-zeolite monolithic catalysts[J]. Chemical Engineering Science, 2013, 87(2):51–66.

[18] Depcik C, Srinivasan A. One + One-Dimensional Modeling of Monolithic Catalytic Converters[J]. Chemical Engineering &Technology, 2011, 34(12):1949–1965.

[19] Braun J., Kurpejovic E., Resch A. et al Efficient Development and Validation Method for SCR Systems[J]. MTZ worldwide, 2018, 79(3):46–51.

[20] HAN Z., REITZ R. D. Turbulence Modeling of Internal Combustion Engines Using RNG *k*−*ε* Models[J]. Combustion Science & Technology, 1995, 106(4–6):267–295.

[21] Liu A B, Mather D, Reitz R D. Modeling the effects of drop drag and breakup on fuel sprays[J]. NasaSti/recon Technical Report N, 1993, 93.

[22] Capetillo A. Ibarra F. Multiphase injector modeling for automotive SCR systems: A full factorial design of experiment and optimization[J]. Computers & Mathematics with Applications, 2017, 74(1):188–200.

[23] Huh, K. Y., and Gosman, A. D., A Phenomenological model of diesel spray atomization[C] Proceedings of the International Conference of Multi-Phase Flows, Sep. 24–27, Tsukuba, Japan, 1991, pp. 515–518.

[24] Pant A, Schmieg S J. Kinetic Model of NO_x_ SCR Using Urea on Commercial Cu−Zeolite Catalyst[J]. Industrial & Engineering Chemistry Research, 2011, 50(9):5490–5498.

[25] Zhang Lixiong, Zhang Li, Jing Xiaojun, et al Emission difference between cold start and hot start WHTC cycles based on a SCR diesel engine[J]. Small Internal Combustion Engine and Motorcycle, 2016, 45 (3): 37–40.

[26] Tong Dehui, Li Guoxiang, et al Design of SCR Converter for Heavy-Duty Diesel Engine Using CFD Technology [J]. Transactions of CSICE, 2008, (6): 20–25.

[27] Tan Piqiang, Du Jiazhen, Hu Zhiyuan, et al The key operation parameters influence on the performance of SCR system of diesel engine [J]. Journal of chemical industry and engineering, 2014, 65 (10): 4063–4070.

[28] Chen Hao, Xie Bin, Ma Jinqiu, et al NOx emission of biodiesel compared to diesel: Higher or lower?[J]. Applied Thermal Engineering, 2018, 137 (1): 584–593.

